# Pectoral muscle mass is not a robust prognostic factor for survival after left ventricular assist device (LVAD) implantation

**DOI:** 10.1186/s13019-024-02547-8

**Published:** 2024-02-09

**Authors:** Freya Sophie Jenkins, Jan-Philipp Minol, Tarik Akar, Esma Yilmaz, Moritz Benjamin Immohr, Ismail Dalyanoglu, Bernhard Korbmacher, Joel Aissa, Udo Boeken, Artur Lichtenberg, Payam Akhyari, Hannan Dalyanoglu

**Affiliations:** 1 Department of Cardiac Surgery, Medical Faculty, Heinrich Heine University, Düsseldorf, Germany; 2 Medical Faculty, Semmelweiss University, Budapest, Hungary; 3 Institute of Diagnostic and Interventional Radiology, Medical Faculty, Heinrich Heine University, Düsseldorf, Germany; 4 Department of Cardiac Surgery, University Hospital Düsseldorf, Moorenstr. 5, 40225 Düsseldorf, Germany

**Keywords:** Left ventricular heart failure, Left ventricular assist device, Pectoral muscle mass, Frailty

## Abstract

**Background:**

Left ventricular assist devices (LVAD) are an established treatment for end-stage left ventricular heart failure. Parameters are needed to identify the most appropriate patients for LVADs. This study aimed to evaluate pectoral muscle mass and density as prognostic parameters.

**Methods:**

This single-center study included all patients with LVAD implantation between January 2010 and October 2017 and a preoperative chest CT scan. Pectoral muscle mass was assessed using the Pectoralis Muscle Index (PMI, surface area indexed to height, cm^2^/m^2^) and pectoral muscle density by Hounsfield Units (HU). Overall mortality was analyzed with Kaplan–Meier survival analysis and 1-year and 3-year mortality with receiver operating characteristic (ROC) curves and Cox regression models.

**Results:**

57 patients (89.5% male, mean age 57.8 years) were included. 64.9% of patients had end-stage left ventricular failure due to ischemic heart disease and 35.1% due to dilated cardiomyopathy. 49.2% of patients had preoperative INTERMACS profile of 1 or 2 and 33.3% received mechanical circulatory support prior to LVAD implantation. Total mean PMI was 4.7 cm^2^/m^2^ (± 1.6), overall HU of the major pectoral muscle was 39.0 (± 14.9) and of the minor pectoral muscle 37.1 (± 16.6). Mean follow-up was 2.8 years (± 0.2). Mortality rates were 37.5% at 1 year and 48.0% at 3 years. Neither PMI nor HU were significantly associated with overall mortality at 1-year or 3-year.

**Conclusions:**

The results of our study do not confirm the association between higher pectoral muscle mass and better survival after LVAD implantation previously described in the literature.

## Background

Left ventricular assist device (LVAD) implantation is generally recommended as a therapeutic option for patients with advanced left ventricular heart failure and shows improved survival rates, better quality of life, and increased functional capacity compared to medical therapy alone [1–4]. Although the operative risks of LVAD are well established, prognostic factors that influence long-term postoperative outcome are less well understood. Several risk factors for mortality after LVAD have been identified, including age, previous cardiac surgery, poor nutrition, hematologic abnormalities, and markers of end-organ or right ventricular dysfunction [5, 6].

Reduced muscle mass is a well-recognized consequence of ageing and has been reported to be associated with poorer outcomes in various disease states [7]. Loss of skeletal muscle mass is common in patients with chronic heart failure and has been shown to be an independent predictor of survival regardless of type of therapy, possibly as a correlate of more advanced disease, frailty, and low exercise capacity [8–10]. Indeed, patients with advanced left ventricular failure have been shown to gain both skeletal muscle mass and weight 6 months after LVAD implantation [11].

Some studies have suggested that low muscle mass is a predictor of outcome after LVAD implantation, with different muscle groups considered as possible indicators of overall muscle mass and thus frailty (e.g., psoas, erector spinae, pectoral muscles) [12–14]. However, results to date are not consistent, and it is not yet clear whether reduction of muscle mass is a robust and independent predictor of long-term outcome after LVAD, or whether reported findings are generalizable across centers.

It has been suggested that pectoral muscle mass may be a better surrogate of frailty than psoas and erector spinae muscles in end-stage disease when patients are less ambulatory [15]. A study published in 2017 by Teigen et al*.* reported a significant reduction in risk of all-cause mortality after LVAD implantation with a higher preoperative pectoralis muscle index (a measure of muscle quantity) and a higher pectoralis muscle Hounsfield unit (a measure of muscle density) [15]. A further study of 64 patients showed that low pectoralis muscle mass increased the risk of 2-year mortality after LVAD [16].

Identifying robust prognostic factors for outcome after LVAD is important to guide patient selection in the clinical setting. The purpose of the current study was to evaluate whether preoperative pectoral muscle mass and density are prognostic factors for overall mortality and 1- and 3- year survival after LVAD implantation at our institution.

## Methods

### Study patients

Patients who underwent LVAD for end-stage left ventricular failure due to ischemic heart disease (IHD) or dilated cardiomyopathy at our institution from January 2010 to October 2017 were included. Patients were required to have an available preoperative chest computed tomography (CT) scan to be eligible for inclusion.

No formal sample size calculation was performed. It was estimated that an acceptable number of patients would be available for analysis based on the inclusion criteria.

### Study design

The single-center study was of retrospective design. All patient data were available and retrievable prior to the start of the study and were anonymized and organized in numerical order for analysis.

### Ethics

The study followed the principles of the Declaration of Helsinki and was approved by the Ethics Committee of Heinrich Heine University, Dusseldorf (study number: 2020–832).

### Radiological evaluation

All preoperative CT examinations were performed with contrast medium. This is in keeping with the methodology described by Kinsey et al. [17], which was followed by Teigen et al. in their study. The Pectoralis Muscle Index (PMI) and Hounsfield Unit (HU) measurements of the major and minor pectoralis muscles were carried out at the level of the aortic arch, to allow comparability with the study by Teigen and colleagues [15]. PMI is the cross-sectional area of the pectoralis muscle in cm^2^ divided by the height squared in m^2^ with HU measuring the density of the pectoralis muscle calculated using the radiological HU value. In all patients pectoral muscle mass and density were measured unilaterally on the right side.

### Data analysis

Statistical significance was established as *p* < 0.05. Long-term outcomes were chosen in the analysis plan based on likely clinical relevance to the study objective. Mortality at 1 and 3 years post LVAD implantation was evaluated by raw mortality rates and overall mortality by Kaplan–Meier survival analysis. Raw mortality rates included all patients that reached the timepoint, with no censoring for heart transplantation. The Kaplan–Meier analysis provides a different survival view as data were censored for heart transplantation. In the Kaplan–Meier survival analysis, the Log-rank test was used for group comparisons.

The effect of variables on mortality at 1 and 3 years was evaluated by receiver operating characteristic (ROC) curves with the corresponding area under the curve (AUC) analyzed by the Mann–Whitney U test. For a valid ability to discriminate the respective measurement variable, the AUC must deviate significantly from AUC = 0.5. The impact of preoperative variables on 1- and 3-year mortality after LVAD implantation was also examined using Cox regression analysis, with a multivariate analysis used to evaluate the impact of variables PMI, pectoralis major area and HU, pectoralis minor area and HU, and clinically relevant survival determinants (age, sex, underlying disease and preoperative extracorporeal membrane oxygenation [ECMO]). Mean values of pectoral muscle parameters in those patients who died And those who survived 1 and 3 years after surgery were compared using the Student's t test. Analysis and evaluation of data were carried out using the statistical software R, version 4.2.0 (R Foundation for Statistical Computing, Vienna, Austria).

## Results

### Study patients

A total of 57 patients were included in the analysis. Table 1 shows the preoperative characteristics of patients prior to LVAD implantation. For pectoralis muscle parameters, mean PMI was 4.7 cm^2^/m^2^ (± 1.6), with a mean HU of 39.0 (± 14.9) for pectoralis major and 37.1 (± 16.6) for pectoral minor. All patients had a preoperative left ventricular ejection fraction (LVEF) < 30%, and an INTERMACS score of 4 or less.

**Table 1:** Preoperative characteristics of the cohort

	**N = 57**
Sex, n (%)	
male	51 (89%)
female	6 (11%)
Age in years, mean (± SD)	57.79 (± 11.75)
Height in cm, mean (± SD)	176 (± 9.0)
Weight in kg, mean (± SD)	80.9 (± 14.4)
Body mass index in kg/m^2^, mean (± SD)	26.20 (± 4.5)
INTERMACS score, n (%)	
1	16 (28%)
2	12 (21%)
3	11 (19%)
4	18 (32%)
Etiology of left ventricular failure, n (%)	
Dilated cardiomyopathy	20 (35%)
Ischemic heart disease	37 (65%)
Past medical history, n (%)	
Arterial hypertension	36 (63%)
Coronary heart disease	36 (63%)
Defibrillator implant	30 (53%)
Myocardial infarct	27 (47%)
PTCA	26 (46%)
Carotid stents	22 (39%)
Hyperlipoproteinemia	21 (37%)
Cardiac surgery	19 (33%)
Diabetes mellitus	19 (33%)
COPD	13 (23%)
Nicotine abuse	13 (23%)
Syncope	5 (9%)
Peripheral vascular disease	4 (7%)
Pacemaker	4 (7%)
Pulmonary emboli	4 (7%)
Dialysis	3 (5%)
Main stem stenosis	2 (4%)
Cerebrovascular disease	1 (2%)
Mechanical circulatory support	
None	38 (67%)
ECLS only	9 (16%)
ECLS, IABP	3 (5%)
IABP only	4 (7%)
Impella only	3 (5%)

Preoperative characteristics of the cohort

*COPD* Chronic obstructive pulmonary disease, *PTCA* Percutaneous transluminal coronary angioplasty, *ECLS* Extracorporeal life support, *IABP* Intraarterial balloon pump

Of the 57 patients, 37 (65%) were admitted as an emergency, and the remaining 20 patients (35%) were transferred from another clinic or admitted electively for LVAD implantation. 28.1% had preoperative critical cardiogenic shock (INTERMACS 1) and 21.1% had progressive decline with increased inotrope dependency and worsening end-organ function (INTERMACS 2), with 33% receiving mechanical circulatory support prior to LVAD implantation. 16 patients (28%) required inotropic medication of which half (8 patients) were invasively ventilated preoperatively. A total of 34 patients (60%) were in sinus rhythm before surgery. Preoperative renal function was normal in 23 patients (40%), with a mean total serum creatinine across the cohort of 1.62 mg/dL (SD ± 0.77), with creatinine values ranging from 0.8 to 4.9 mg/dL.

The therapeutic objective of LVAD was Bridge to Transplant (BTT) in the majority of patients (70%), with the goal of Bridge to Recovery (BTR) in 1 patient (2%) and Destination Therapy (DT) in 16 patients (28%). The majority of patients (72%) received a Heartware LVAD implantation, with 26% receiving a Heartmate III, and 2% a Heartmate II device.

In our cohort 30 patients had an implantable cardioverter-defibrillator (ICD), all of which were on the left side of the body. As all the pectoral muscle measurement were performed on the right side, we do not expect any influence of the indwelling ICD. In terms of mechanical circulatory support, in the time period included in our publication, none of the mechanical circulatory support devices were implanted via the axillary artery as this practice was not yet established at our institution.

### Survival analysis

Patients were followed for a mean of 2.8 years (± 0.2). Mortality rates were 37.5% at one year and 48.0% at three years. Not all deaths after 1 and 3 years were cardiac-related. Within a year (excluding those who died in hospital or received a heart transplant) 3 had massive intracerebral hemorrhage, 2 had septic shock, 2 died as a result of uncontrollable arrhythmias, and 2 died of treatment refractory cardiogenic shock. Another 2 patients died within 3 years while the LVAD was running (one with massive intracerebral hemorrhage and one who died at a care facility with cause of death unknown).

**Fig. 1:**
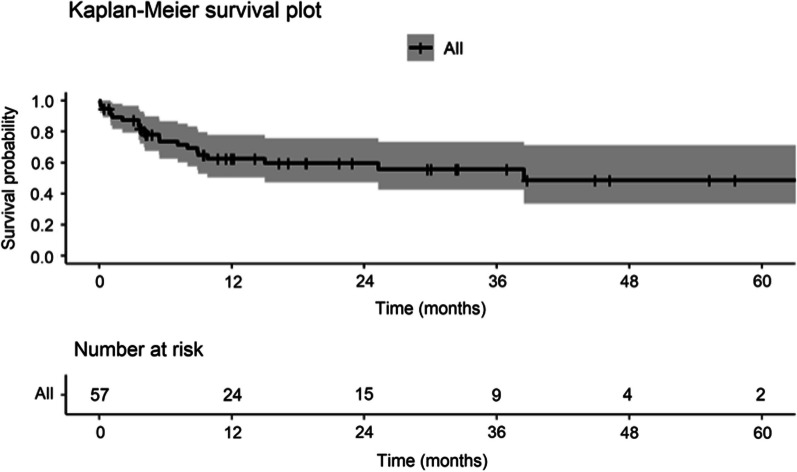
Kaplan–Meier Survival for Overall Cohort (n = 57, median age 57.6 years). Abbreviations: LVAD = Left ventricular assist device. Patient data censored at heart transplantation

The overall survival curve for the cohort is shown in Fig. 1. As expected the highest mortality rate was observed in the first year after LVAD implantation.

**Fig. 2:**
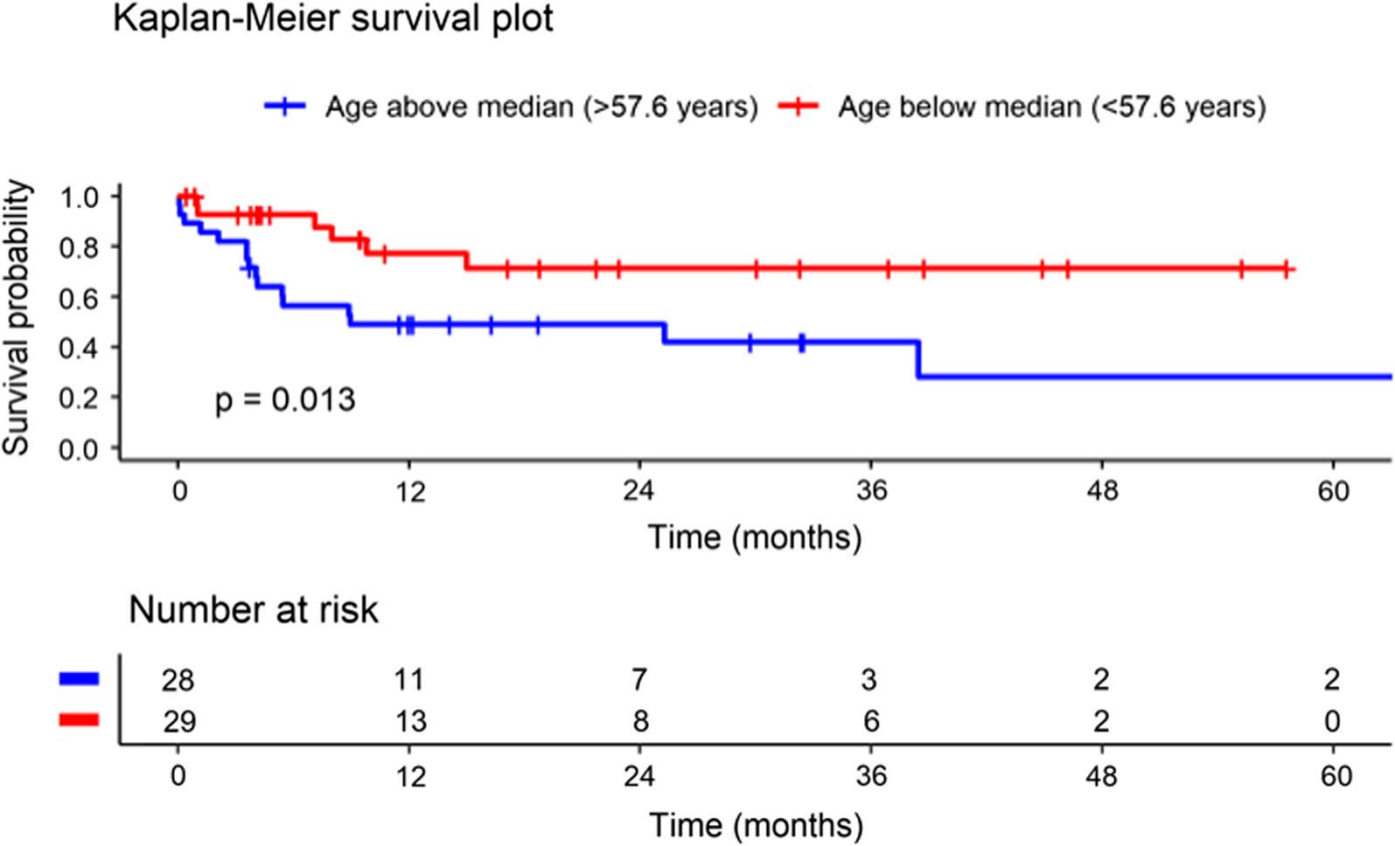
Kaplan–Meier Survival Curve by Age (Log-rank test used for group comparisons). Abbreviations: LVAD = Left ventricular assist device. Patient data censored at heart transplantation

Younger patients (age below the median, n = 29) had significantly better survival than older patients (age above the median, n = 28) (*p* = 0.013) (Fig. 2). No significant differences were observed for the survival curves for patients with left ventricular failure due to IHD or due to dilated cardiomyopathy (*p* = 0.58) or by INTERMACS score (*p* = 0.32).

**Fig. 3:**
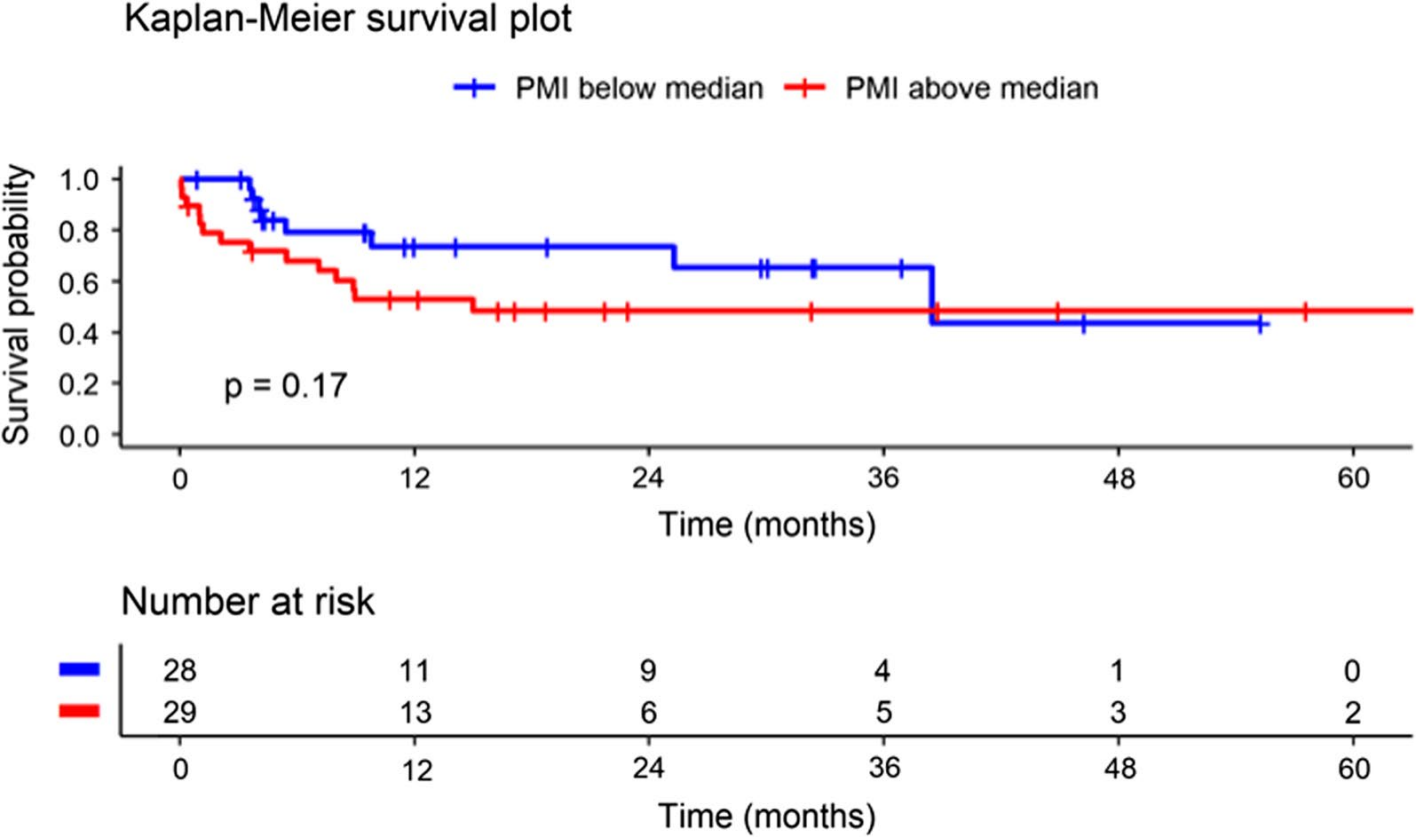
Kaplan–Meier Survival Curve by Preoperative PMI (Log-rank test used for group comparisons). Abbreviations: LVAD = Left ventricular assist device. Patient data censored at heart transplantation

There was a trend to better survival in patients with lower pectoral muscle mass (PMI below the median, n = 28) compared to patients with higher pectoral muscle mass (PMI above the median, n = 29), with consistent divergence of Kaplan–Meier survival curves over time (Fig. 3).

**Fig. 4:**
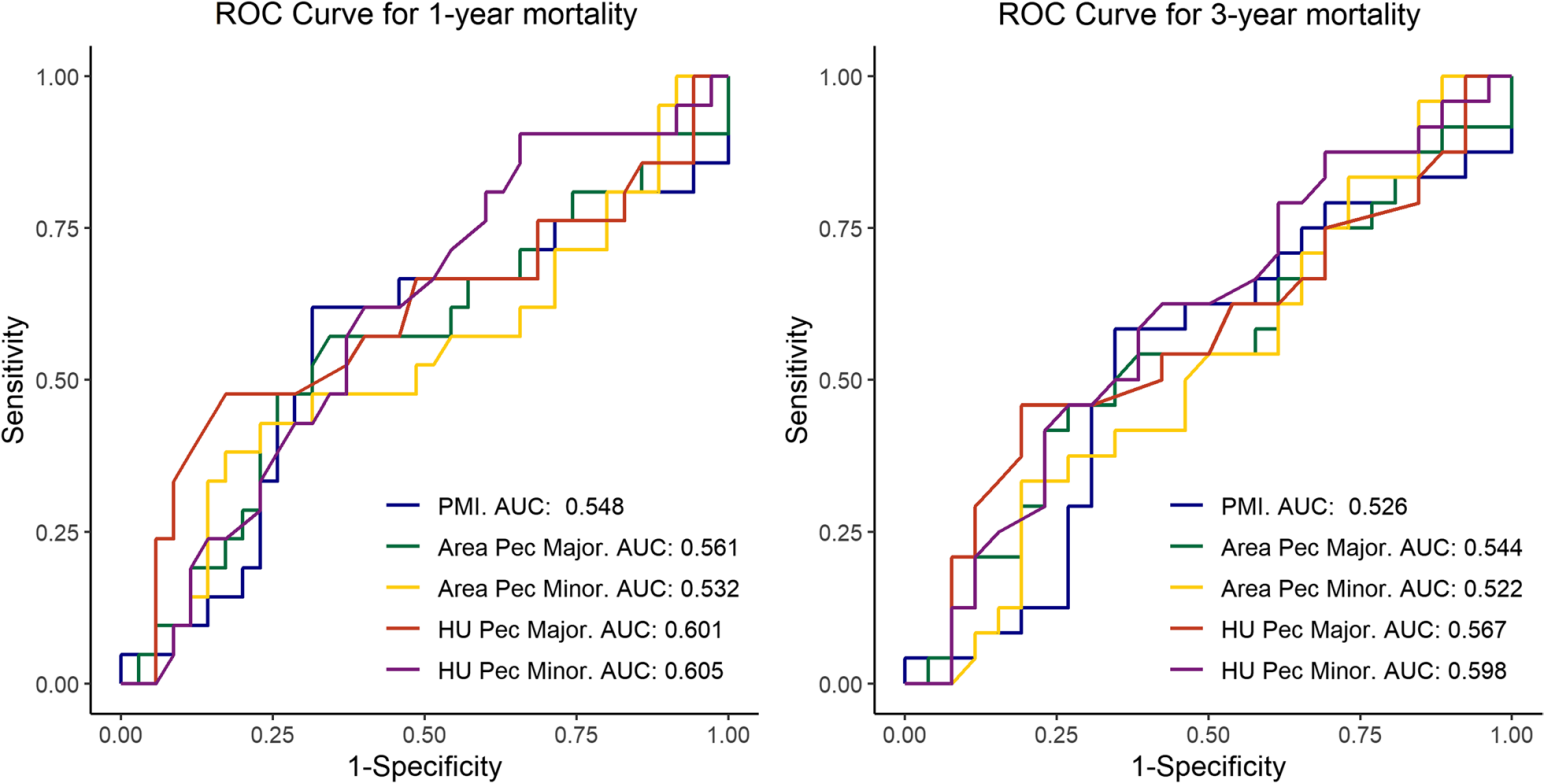
Receiver Operating Characteristic Curves for 1- and 3-year Mortality. Abbreviations: AUC = Area under the curve; PMI = Pectoralis Muscle Index; HU = Hounsfield Unit

Figure 4 shows the ROC curves for 1- and 3-year mortality, and Table 2 shows the analysis of the AUC of the ROC curve for the parameters of PMI, pectoral major and pectoral minor area, or pectoral major and minor density. There was no significant difference in the AUC from 0.5 for any of the pectoral mass or density parameters, such that these cannot be considered valid discriminators of 1- and 3- year mortality in the current study. In both the univariate and multivariate Cox regression analysis (Table 3), there was no significant relationship with 1- and 3-mortality after LVAD for any of the pectoral muscle mass or density parameters examined. In the multivariate analysis there was also no significant relationship with 1- and 3- year mortality for sex, underlying disease, or preoperative ECMO, but a significant relationship was observed with mortality at 3 years for patient age (*p* = 0.045).

**Table 2:** Area Under the Receiver Operating Characteristic Curve for Pectoral Mass Parameters

Variable	1-Year Mortality		3-Year Mortality	
	AUC	*p*-value*	AUC	*p*-value*
PMI	0.548	0.28	0.526	0.38
Pectoralis major area	0.561	0.23	0.544	0.30
Pectoralis minor area	0.532	0.35	0.522	0.40
Pectoralis major HU	0.601	0.11	0.567	0.21
Pectoralis minor HU	0.605	0.10	0.598	0.12

Area Under the Receiver Operating Characteristic Curve for Pectoral Mass Parameters

*PMI* Pectoral muscle index, *HU* Hounsfield unit

^*^Mann–Whitney-U-Test (Null-hypothesis, AUC = 0.5)

**Table 3:** Cox Regression Analysis of Preoperative Pectoral Mass Variables on Mortality

	1-Year Mortality			3-Year Mortality		
Variable	B	SE	*p*-value	B	SE	*p*-value
*Univariate Analysis*						
PMI	0.100	0.176	0.57	0.046	0.176	0.80
Pectoralis Major area	0.038	0.072	0.60	0.021	0.073	0.77
Pectoralis Minor area	0.062	0.175	0.72	0.025	0.171	0.88
HU Pectoralis Major	− 0.021	0.019	0.27	− 0.014	0.019	0.45
HU Pectoralis Minor	− 0.021	0.018	0.23	− 0.021	0.018	0.24
*Multivariate Analysis*						
PMI	0.163	0.208	0.43	0.123	0.214	0.57
Age	0.039	0.031	0.21	0.069	0.035	0.045
Sex	− 1.387	1.193	0.25	− 0.295	1.062	0.78
Underlying disease	0.026	0.698	0.97	− 0.240	0.699	0.73
Preoperative ECMO	− 1.265	0.898	0.16	− 0.370	0.880	0.67
Pectoralis major area	0.067	0.092	0.47	0.070	0.096	0.47
Age	0.040	0.031	0.21	0.070	0.035	0.042
Sex	− 1.255	1.253	0.32	− 0.096	1.140	0.93
Underlying disease	0.059	0.700	0.93	− 0.200	0.701	0.78
Preoperative ECMO	− 1.268	0.900	0.16	− 0.428	0.889	0.63
Pectoralis minor area	0.134	0.200	0.50	0.060	0.198	0.76
Age	0.038	0.032	0.23	0.069	0.035	0.049
Sex	− 1.519	1.170	0.19	− 0.404	1.037	0.70
Underlying disease	0.013	0.696	0.99	− 0.249	0.699	0.72
Preoperative ECMO	− 1.285	0.907	0.16	− 0.310	0.874	0.72
Pectoralis major HU	− 0.028	0.021	0.18	− 0.021	0.021	0.31
Age	0.045	0.034	0.19	0.074	0.037	0.04
Sex	− 1.805	1.188	0.13	− 0.603	1.053	0.57
Underlying disease	− 0.277	0.749	0.71	− 0.471	0.749	0.53
Preoperative ECMO	− 1.178	0.899	0.19	− 0.312	0.867	0.72
Pectoralis minor HU	− 0.026	0.020	0.19	− 0.020	0.019	0.28
Age	0.036	0.032	0.27	0.067	0.036	0.06
Sex	− 1.826	1.206	0.13	− 0.636	1.077	0.56
Underlying disease	− 0.105	0.725	0.88	− 0.376	0.731	0.61
Preoperative ECMO	− 1.290	0.908	0.16	− 0.335	0.873	0.70

Cox Regression Analysis of Preoperative Pectoral Mass Variables on Mortality

*B* Beta coefficient (Regressions-coefficient), *SE* Standard error, *PMI* Pectoral muscle index, *HU* Hounsfield unit, *ECMO* Extracorporeal membrane oxygenation

No significant differences in mean values for any of the pectoral muscle variables by survival status were observed at 1 year and 3 years after LVAD implantation (Table 4).

**Table 4:** Comparison of Pectoral Muscle Parameters for Patients Dead or Alive at 1 and 3 Years Post LVAD Implantation

1 year post LVAD implantation				
	Dead (n = 21)	Alive (n = 35)		
Variable	Mean	Mean	Student's *t*	*p*-value
PMI	4.873	4.626	− 0.54	0.59
Pectoralis Major area	11.235	10.669	− 0.52	0.61
Pectoralis Minor area	3.808	3.653	− 0.35	0.73
HU Pectoralis Major	36.238	40.800	1.08	0.29
HU Pectoralis Minor	33.429	38.943	1.26	0.22

3 years post LVAD implantation				
	Dead (n = 24)	Alive (n = 26)		
Variable	Mean	Mean	Student's *t*	*p*-value
PMI	4.833	4.714	− 0.26	0.80
Pectoralis Major area	11.194	10.873	− 0.28	0.78
Pectoralis Minor area	3.724	3.655	− 0.15	0.89
HU Pectoralis Major	36.750	40.077	0.75	0.46
HU Pectoralis Minor	33.083	38.769	1.19	0.24

Comparison of Pectoral Muscle Parameters for Patients Dead or Alive at 1 and 3 Years Post LVAD Implantation. Follow-up was not achieved for 1 patient at 1 year and 7 patients at 3 years due to the end of the study

*PMI* Pectoralis muscle index, *HE* Hounsfield unit

## Discussion

The study showed no significant association between pectoral muscle mass or density and 1-year or 3-year mortality after LVAD implantation at our institution, challenging the robustness and generalizability of pectoral muscle parameters as a prognostic factor after LVAD implantation.

As expected, in the current study, younger patients had significantly better survival than older patients, but no significant differences were observed for survival curves for patients with IHD or dilated cardiomyopathy or by INTERMACS score. Although it did not reach statistical significance and thus the null hypothesis cannot be rejected, there was a trend toward better survival in patients with lower pectoral muscle mass compared to those with higher pectoral muscle mass, with consistent divergence of the Kaplan–Meier survival curves over time.

Analysis of the ROC curves also showed that the pectoral muscle parameters evaluated do not appear to be valid discriminators of 1- and 3- year mortality in the current study. This finding is supported by the Cox regression analysis that did not show a significant relationship with 1- and 3-mortality after LVAD for any of the pectoral muscle parameters examined. As expected, the regression analysis confirmed a significant relationship between younger age and better survival at 3 years. No significant differences in mean values for any of the pectoral muscle variables by survival status were observed at 1 and 3 years after LVAD implantation.

The results of the current study contrast with other studies that have suggested that low preoperative pectoral muscle mass predicts decreased survival after LVAD [15, 16].

Reasons for the different findings between studies are not clear, but the patient population in the current study are sicker than those reported by Teigen et al*.*, given that considerably more patients in our study had an INTERMACS score of 1 (28% vs 8% in the Teigen study). In keeping with our patients being sicker, muscle mass in our study is lower than that reported in the Teigen study (lowest tertile mean PMI in our study 3.3 vs. Teigen 3.5; middle tertile 4.5 vs. Teigen 5.3; highest tertile 6.5 vs. Teigen 7.9).

A potential explanation for why pectoral muscle mass does not appear to be a robust prognostic factor in patients with end-stage left ventricular failure and why lower pectoral muscle mass is associated with better survival in the current study is that the sickest patients are bed bound and have to use their arms more to move compared to less sick ambulatory patients. Therefore, in the sickest patients, the pectoral muscles are stronger than would be expected by their general condition. Obviously once patients have an INTERMACS score of 1 and 2 they are unlikely to be using their arms to any extent. However many of our cohort were admitted from home and from other hospitals with recent decompensation of their left ventricular heart failure. Prior to decompensation, the hypothesis regarding bed bound patients using their arms would still be valid. Our hypothesis suggests that measuring muscle mass other than the pectoral muscles may be more appropriate when determining prognostic factors after LVAD.

Muscle mass of psoas and erector spinae have been suggested as surrogates for frailty and thus prognostic factors for outcome after LVAD implantation. One study in 32 patients showed a significant association between low preoperative psoas muscle area and reduced short-term outcome after LVAD implantation (composite endpoint of hospital mortality and prolonged hospital stay > 30 days), but showed no association with overall mortality [13]. Another study in 20 patients showed significantly higher 30-day mortality after LVAD implantation in those with low psoas muscle area, and a recent study in our own institution showed that psoas muscle index was a significant predictor of one-year mortality after LVAD implantation [14].

A study in 119 patients undergoing LVAD implantation reported that preoperative erector spinae mass showed a weak but significant negative correlation with duration of hospital stay but no correlation with major adverse cardiovascular events, in-hospital mortality, or long-term survival [12].

However, muscle mass measured in other muscles may not be practical (e.g. CT scans of psoas muscle are not routinely performed prior to LVAD), muscles of the abdominal regions are more susceptible to edema, and spinal muscles may not be an accurate measure of general muscle mass and frailty as upright posture is not maintained in end-stage disease.

The current study provides new information on the use of pectoral muscle mass and density parameters as prognostic factors after LVAD implantation and suggests that low pectoral muscle mass and density may not be associated with reduced survival in all cohorts of patients in all centers. More studies are required, including prospective studies before pectoral muscle mass and density can be considered robust and generalizable prognostic factors before LVAD implantation.

### Limitations

The study has some limitations, including a relatively small sample size, potential impact of device diversity on mortality, requirement to have an available preoperative chest CT scan, and the known limitations associated with retrospective studies. The relatively small sample size and censoring at heart transplantation resulted in a very low number of patients being at risk in the Kaplan–Meier analysis by 3 years post LVAD implantation (n = 9). However, the number still at risk at 1 year was 25, which should allow meaningful conclusions from the data at least to 1 year post LVAD implantation. In the raw mortality analysis 35 patients were alive at 1 year and 26 patients were alive at 3 years.

Device diversity has the potential to influence the outcome of death. In a secondary analysis of our study we observed no differences in pectoral muscle mass and mortality between the devices (p = 0.89 for overall mortality) but numbers in the non-Heartware groups are small such that robust conclusions about the influence of device type on mortality after LVAD cannot be made from the current study.

High mortality rates in retrospective studies have the potential to bias results. The 1-year mortality in our cohort (37.5%) is slightly higher compared to the results of the EUROMACS registry (30%) [18]. The requirement for a CT scan in our study likely resulted in sicker patients being included, which would in turn result in higher mortality rates. In addition our study population has important differences compared to the EUROMACS registry that would explain the differences in 1-year mortality rates. For example, our cohort included more patients with LVAD as ‘Destination therapy’ (28.1%) vs EUROMACS (around 20%), with 1-year mortality for high-risk ‘Destination therapy’ patients in our study of 43.8%. Our cohort also had a higher rate of transplantation in the first year (17.5% vs 7.5% in EUROMACS).

## Conclusions

Treatment of end-stage left ventricular heart failure remains challenging. Prognostic factors for long-term survival after LVAD implantation are urgently needed to select appropriate patients. In the current study, we evaluated pectoral muscle mass as an easily measurable parameter of frailty in this context. The results of our study suggest that pectoral muscle mass or density are not a robust prognostic factors for survival after LVAD implantation in all cohorts of patients. Consequently, more studies are needed to evaluate additional factors and improve prognostic models for the outcome after LVAD implantation.

## Data Availability

The datasets used and/or analyzed during the current study are available from the corresponding author on reasonable request.

## Abbreviations

AUC: Area under the curve
BTR: Bridge to recovery
BTT: Bridge to transplant
CT: Computed tomography
DCM: Dilated cardiomyopathy
DT: Destination therapy
ECMO: Extracorporeal membrane oxygenation
HU: Hounsfield Unit
ICD: Implantable cardioverter-defibrillator
IHD: Ischemic heart disease
LVAD: Left ventricular assist device
LVEF: Left ventricular ejection fraction
PMI: Pectoralis muscle index
ROC: Receiver operating characteristic
